# Influence of acquired obesity on coronary vessel wall late gadolinium enhancement in discordant monozygote twins

**DOI:** 10.1007/s00330-016-4616-8

**Published:** 2016-10-14

**Authors:** Marcus R. Makowski, Christian H. P. Jansen, Ullrich Ebersberger, Tobias Schaeffter, Reza Razavi, Massimo Mangino, Tim D. Spector, Rene M. Botnar, Gerald F. Greil

**Affiliations:** 1 Division of Imaging Sciences and Biomedical Engineering, King’s College London, London, SE1 7EH UK; 2 Wellcome Trust and EPSRC Medical Engineering Centre, London, UK; 3 BHF Centre of Excellence, King’s College London, London, UK; 4 NIHR Biomedical Research Centre, King’s College London, London, UK; 5 Department of Radiology, Charité-Universitätsmedizin, Berlin, Germany; 6 Department of Cardiology and Intensive Care Medicine, Heart Center Munich-Bogenhausen, Munich, Germany; 7 Department of Twin Research and Genetic Epidemiology, King’s College London, London, UK; 8 National Institute for Health Research (NIHR) Biomedical Research Centre at Guy’s and St. Thomas’ Foundation Trust, London, UK

**Keywords:** MRI, 3D-IR-TFE, Vessel wall scan, Coronary, Twin

## Abstract

**Objectives:**

The aim of this study was to investigate the impact of BMI on late gadolinium enhancement (LGE) of the coronary artery wall in identical monozygous twins discordant for BMI. Coronary LGE represents a useful parameter for the detection and quantification of atherosclerotic coronary vessel wall disease.

**Methods:**

Thirteen monozygote female twin pairs (n = 26) with significantly different BMIs (>1.6 kg/m2) were recruited out of >10,000 twin pairs (TwinsUK Registry). A coronary 3D-T2prep-TFE MR angiogram and 3D-IR-TFE vessel wall scan were performed prior to and following the administration of 0.2 mmol/kg of Gd-DTPA on a 1.5 T MR scanner. The number of enhancing coronary segments and contrast to noise ratios (CNRs) of the coronary wall were quantified.

**Results:**

An increase in BMI was associated with an increased number of enhancing coronary segments (5.3 ± 1.5 vs. 3.5 ± 1.6, *p* < 0.0001) and increased coronary wall enhancement (6.1 ± 1.1 vs. 4.8 ± 0.9, *p* = 0.0027) compared to matched twins with lower BMI.

**Conclusions:**

This study in monozygous twins indicates that acquired factors predisposing to obesity, including lifestyle and environmental factors, result in increased LGE of the coronary arteries, potentially reflecting an increase in coronary atherosclerosis in this female study population.

***Key points*:**

*• BMI-discordant twins allow the investigation of the influence of lifestyle factors independent from genetic confounders.*

*• Only thirteen obesity-discordant twins were identified underlining the strong genetic component of BMI.*

*• In female twins, a BMI increase is associated with increased coronary late gadolinium enhancement.*

*• Increased late gadolinium enhancement in the coronary vessel wall potentially reflects increased atherosclerosis.*

**Electronic supplementary material:**

The online version of this article (doi:10.1007/s00330-016-4616-8) contains supplementary material, which is available to authorized users.

## Introduction

Obesity has been recognized as a major contributor to the global burden of chronic disease and disability [1–3]. Epidemiological, clinical, and preclinical studies conducted in the field of cardiovascular medicine have led to significant progress in our understanding of modifiable and non-modifiable risk factors in cardiovascular disease. Despite the availability of cost-effective interventions to reduce risks, the World Health Report 2002 estimated 58 % of diabetes, 21 % of ischemic heart disease and 8–42 % of certain cancers globally were attributable to a body mass index (BMI) above 21 kg/m^2^. The BMI was shown to be associated with coronary atherosclerosis and the risk for cardiovascular events [4]. Although it is recognized that an increase in BMI is associated with an increase in cardiovascular events, this association is highly complex. Currently, it is not well investigated whether or to which extent this association is confounded by genetic factors [4].

Identical twins are matched for genes, age, ethnicity, gender and usually share the same lifestyle and environment [5, 6]. In addition to genetic factors, lifestyle and environmental factors can also lead to obesity, which manifests as an increase in BMI and concurrent metabolic changes. A collective of identical twins discordant for BMI offers the unique opportunity to investigate the influence of these two parameters on the development of atherosclerosis in the coronary vessel wall independent from genetic confounders.

Extravascular gadolinium-based contrast agents are paramagnetic media used in clinical routine for magnetic resonance imaging (MRI). This type of contrast agent was shown to accumulate in atherosclerotic plaques with an increased expression of fibrotic tissue components/extracellular matrix proteins, increased distribution volume, delayed clearance, or increased neovascularization [7–10]. Previous studies have demonstrated that this type of contrast agent can be used for the detection and quantification of atherosclerotic coronary vessel wall disease [8, 10, 11]. In this context, different studies have demonstrated that the extent of enhancement correlates with the severity of atherosclerosis as detected with multislice computed tomography and quantitative coronary angiography [9, 10].

The purpose of this study of a unique study collective of discordant monozygous twins was to investigate to which extent acquired factors predisposing to obesity, including lifestyle and environment, result in increased late gadolinium enhancement of the coronary arteries.

## Methods

### Study population

This project was performed in cooperation with a research group working with the TwinsUK Registry. As part of this cooperation, the TwinsUK Registry was systematically screened for subjects with significantly different BMIs. Thirteen identical twin pairs (n = 26 subjects) with differences in BMI were recruited from more than 10,000 twin pairs from the TwinsUK Registry [12]. As a threshold, a difference in BMI between twin pairs of 1.6 kg/m2 was chosen. The average difference in BMI between the twin pairs in this study was 6.3 ± 4.4 kg/m2. This small study population is due to the rarity of identical twins with significant differences in BMI (thirteen out of more than 10,000) due to the strong genetic contribution and matching lifestyles. The average difference between identical twins is usually less than one kg. The strength of this study, however, is that monozygote twin pairs were investigated. Twins from the registry were recruited from the general population through national media campaigns in the UK. St Thomas' Hospital research ethics committee approved the study (ethics number EC95/041), and informed consent was obtained from participating twins. None of the subjects in this study had a known history of cardiovascular disease or reported symptoms relating to cardiovascular disease. Zygosity was established in all subjects by standardized questionnaire and was confirmed by DNA fingerprinting. Exclusion criteria included: twins younger than 18 years, subjects with mental disorders or unable to give consent, claustrophobia, known allergies or contraindications to gadolinium, pregnancy or breastfeeding subjects, unstable angina pectoris (CCS IV), heart failure (NYHA IV), significant cardiac arrhythmia or impaired renal function (GFR < 30 ml/min).

### Risk factor assessment

Demographics, medical history and medication were available as part of the Twins UK cohort study. Subjects were considered current smokers if they had smoked at least 1 cigarette a day within the last 30 days. Hypertension was defined as systolic blood pressure ≥ 140 mmHg, diastolic blood pressure ≥ 90 mmHg or current use of antihypertensive therapy. Diabetes was defined by self-report, a haemoglobin A_1c_ level ≥6.5 %, or current use of hypoglycemic medication. Hypercholesterolemia was characterized by a fasting serum low-density lipoprotein cholesterol level ≥140 mg/dl on direct measurement or current use of lipid-lowering agents. The Framingham Risk Score (FRS) [13] was used to predict the 10-year risk of coronary heart disease in subjects without overt congenital heart disease. The BMI was calculated as body weight divided by body height squared (kg/m^2^). The Short Form 36 (SF-36) Health Survey questionnaire was used (see supplementary material). Intrapair differences in BMI between twin pairs were on average higher than 2.5 kg/m2. In all twins with an increase in BMI, an increase in FRS was measured. Mean BMI was 29.6 (range 20.1–40.5) versus 35.8 (range 26.3–52.5) and mean FRS was 13.4 (range 5–18) versus 16.4 (range 10–20). For details, please see Table 1.

**Table 1:** Characteristics of the trial participants

Characteristics	Twin group 1	Twin group 2	*p*
	Low BMI	High BMI	
Age - yrs			
Mean	58	58	1
Range	37–70	37–70	
Female - no. (%)	13 (100)	13 (100)	1
Body mass index			
Mean	29.6	35.8	<0.001
Range	20.1–40.5	26.3–52.5	
Framingham risk score			
Mean	13.4	16.4	0.02
Range	5–18	10–20	
Blood pressure - mm Hg			
Systolic			
Mean	137	145	0.19
Range	113–173	120–180	
Diastolic			
Mean	79	86	0.24
Range	65–98	67–107	
Current Ssmoker - no. (%)	0 (0)	1 (8)	
HDL cholesterol - mmol/l			
Mean	1.4	1.4	0.32
Range	0.7–2.3	0.8–1.7	
Total cholesterol - mmol/l			
Mean	5.4	6.5	0.01
Range	2.8–7.3	4.7–7.6	

The mean and range of relevant factors are given for all twins, for the twin group with low BMI and for the twin group with high BMI. Differences between these two groups are also reported.

### MRI imaging and coronary vessel wall imaging

**Fig. 1:**
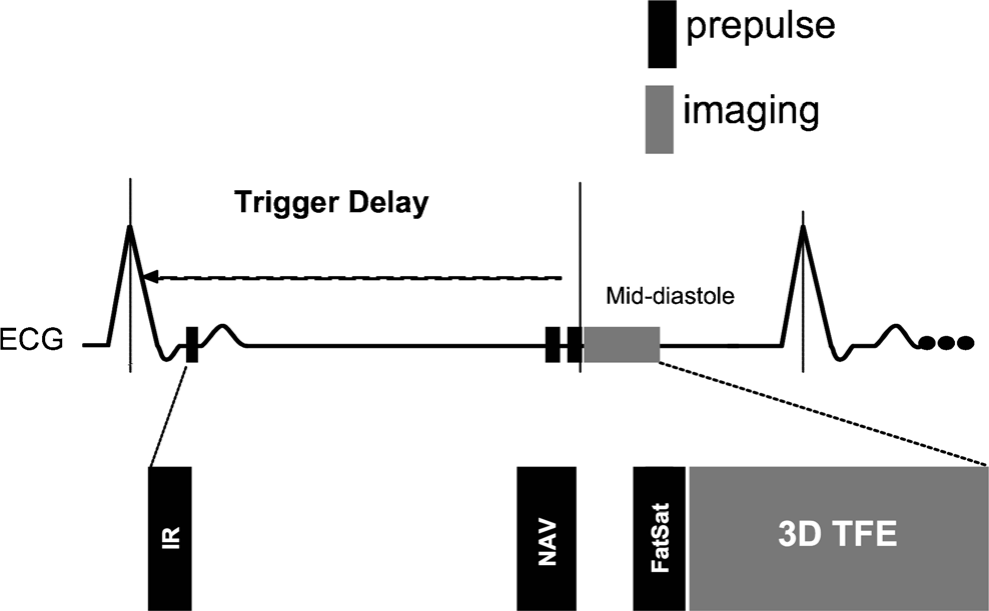
MRI pulse diagram for the visualization and quantification of the contrast agent in the coronary wall. **a** coronary navigator-gated, vector ECG-triggered, fat-suppressed T1-weighted, three-dimensional gradient-echo inversion recovery sequence (3D-IR-TFE) was performed prior to and 30 to 40 minutes after the administration of Gd-DTPA (0.2 mmol/kg). Image acquisition was performed in mid-diastole. Additionally, to compensate for breathing, navigator gating and tracking was performed

MRI examinations were performed using a 1.5-Tesla (T) MR system (Achieva, Philips Healthcare, Best, Netherlands). The system was equipped with high-performance gradients with a maximum amplitude of 30 mT/m and a slew rate of 150 mT/m/msec. All subjects were examined in the supine position with electrocardiographic leads placed on the anterior left hemi thorax for vector ECG triggering. A 32-element cardiac phased-array receiver coil was used. All MRI examinations were performed free-breathing without the use of sedation or general anaesthesia. First, a coronary MR angiography (MRA) was performed with the previously described navigator-gated free-breathing and cardiac-triggered three-dimensional (3D) steady-state free precession sequence [14]. Imaging parameters of the coronary MRA included: a matrix of 256 x 256, a field of view of 320 x 320 mm, and the resulting acquired in-plane resolution was 1.25 x 1.25 mm. The acquired slice thickness was 3 mm and the reconstructed slice thickness was 1.5 mm. The acquisition window was limited 80 to 100 ms. Further parameters included a repetition time/echo time (TR/TE) of 4.2/2.1 ms and a flip angle of 110 degrees. The acquired number of slices was 24. Based on the MRA, a coronary navigator-gated, vector ECG-triggered, fat-suppressed, T1-weighted, 3D gradient-echo inversion recovery late gadolinium enhancement sequence (Fig. 1) was planned. Image acquisition was performed in mid-diastole. To compensate for breathing, navigator gating and tracking with a 5-mm gating window was performed [8, 9, 11, 15]. In all subjects, imaging using a 3D IR TFE was performed prior to and after administration of 0.2 mmol/kg of gadolinium-diethylenetriamine pentaacetic acid. Image parameters of the 3D IR TFE included: spatial resolution 1.25 x 1.25 x 3 mm; 20 slices were acquired; the repetition time was 6.1 ms; the echo time was 1.9 ms; and the flip angle was 30°. The acquisition window was limited to 80–100 ms. The inversion time (TI) was derived from a Look-Locker sequence aiming for maximal suppression of the blood pool signal. Parallel imaging was not applied. Overall scan time was 55 ± 3 minutes as imaging was performed prior to and 30 to 40 minutes following the administration of gadolinium-diethylenetriamine pentaacetic acid [8, 9, 11, 15].

### Data analysis

**Fig. 2:**
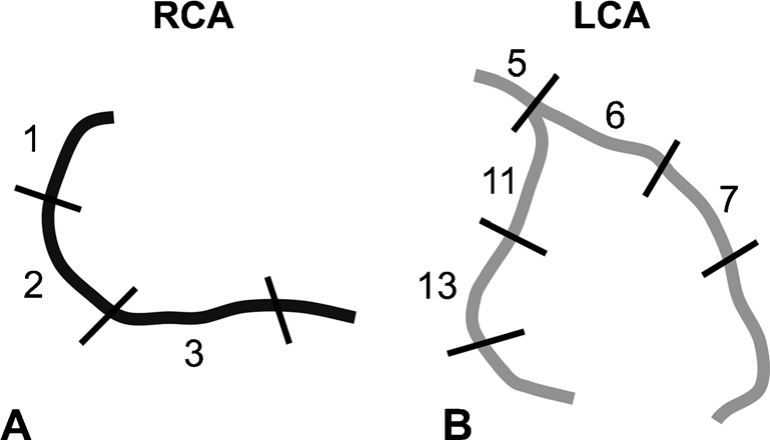
Segmental model for the assessment of coronary enhancement. Coronary arteries were subdivided into eight segments. For an accurate identification of the segments, the segments were pre-defined according to distance from the coronary origin in the aortic root. The right coronary artery (RCA, **a**) was analyzed in 3 segments (*1*–*3*), the left coronary artery (LCA, **b**) within the left main artery (1 segment, *5*), the left anterior descending (2 segments, *6*, *7*), and the circumflex artery (2 segments, *11*, *13*)

**Fig. 3:**
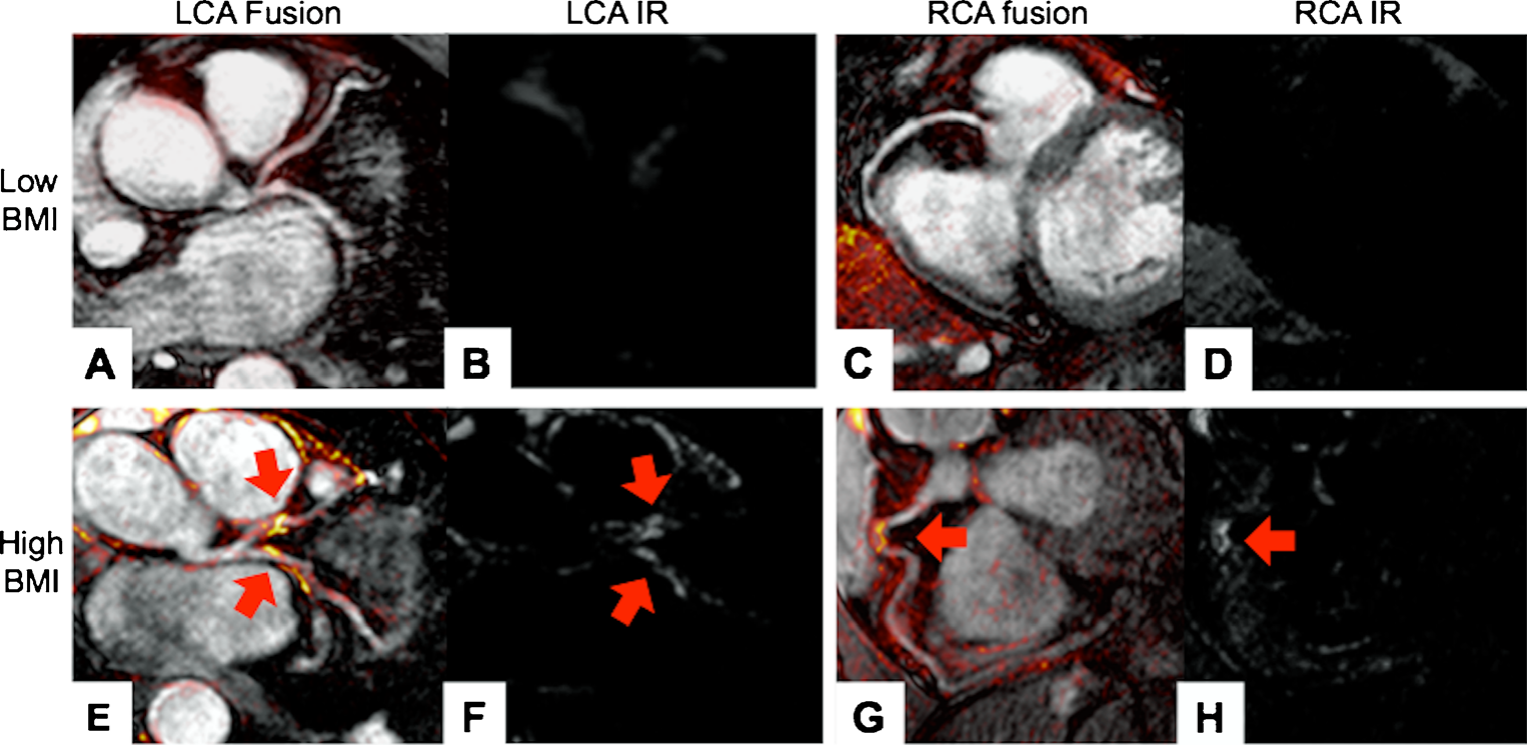
Assessment of coronary enhancement after the administration of Gd-DTPA in monozygous twins. **a**–**d**: Fusion images (**a**, **c**) of coronary MRAs with matched vessel wall scans. Typical example of the enhancement pattern (**b**, **d**) in the left and right coronary artery in twins with a low BMI after administration of the Gd-DTPA. Only a mild signal enhancement can be appreciated in the left and right coronary artery. **e**–**h**: Example of coronary enhancement in twins with a high BMI on fused images (**e**, **g**) as well as inversion recovery images (**f**, **h**). A relatively strong coronary enhancement can be observed in the proximal right and left coronary artery (*red arrow*)

For the analysis of the MRI data sets, the coronary artery tree was broken down into eight segments after a modified American Heart Association (AHA) classification. All segments were analysed individually (Fig. 2) [8, 16, 17]. MR images were reformatted with a semiautomatic vessel analysis tool (Soap-Bubble, release 5.0) [18]. A technique, described by Oakes at al. [19], was adapted for the quantification of coronary vessel wall enhancement with OsiriX version 3.9.1 [20]. Late gadolinium enhancement (LGE) was defined as areas in the vessel wall with a signal intensity higher than two standard deviations compared to the normal non-enhancing vessel wall. With the coronary MRA used as an overlay roadmap (automatic image fusion, Fig. 3), coronary vessel wall enhancement was measured. As the anatomy of the coronary artery tree slightly varies between twin pairs, regions of interest (ROIs) had to be adjusted manually for each twin pair. The variation of size for each segment was less than 10 % between twin pairs. ROIs were exactly copied within twin pairs to enable a high reproducibility, as the anatomical tree did not show variations within twin pairs. To determine the signal intensity (SI) of blood, a contour was drawn in the ascending aorta. Noise was measured in the area anterior to the chest wall by drawing a contour manually. Contrast enhancement within the coronary wall and the aortic wall was defined as the contrast to noise ratio CNR = ((SI_wall_ - SI_blood_)/SD_noise_) [21]. Prior to the analysis, a trial assessment of five separate MR data sets for quality assurance was performed by the two readers together. For the analysis of the data sets, readers were blinded to the clinical information regarding the different twin groups. All data sets were analyzed independently in a blinded and random order. Late gadolinium enhancement measurements were repeated to calculate the intraobserver and interobserver variability. Two observers with >8 years of cardiovascular MRI experience analyzed data in an independent manner.

### Statistical analysis

For statistical analysis, SPSS software (SPSS 17.0, SPS Inc., Chicago, IL, USA) was used. Results were expressed as mean ± SD. The Shapiro–Wilk test and visual confirmation was used to confirm the normal distribution of the data. A Student’s *t* test was applied for the comparison of continuous and discrete variables. Bonferroni correction was used to correct for repeated comparisons. A *p* value < 0.05 was considered to indicate statistical significance.

## Results

### Study population:

A total of thirteen homozygote female twin pairs (median age range 37 to 70 years) were investigated. Patient and baseline characteristics of the study population are summarized in Table 1.

### Coronary vessel wall late enhancement

**Fig. 4:**
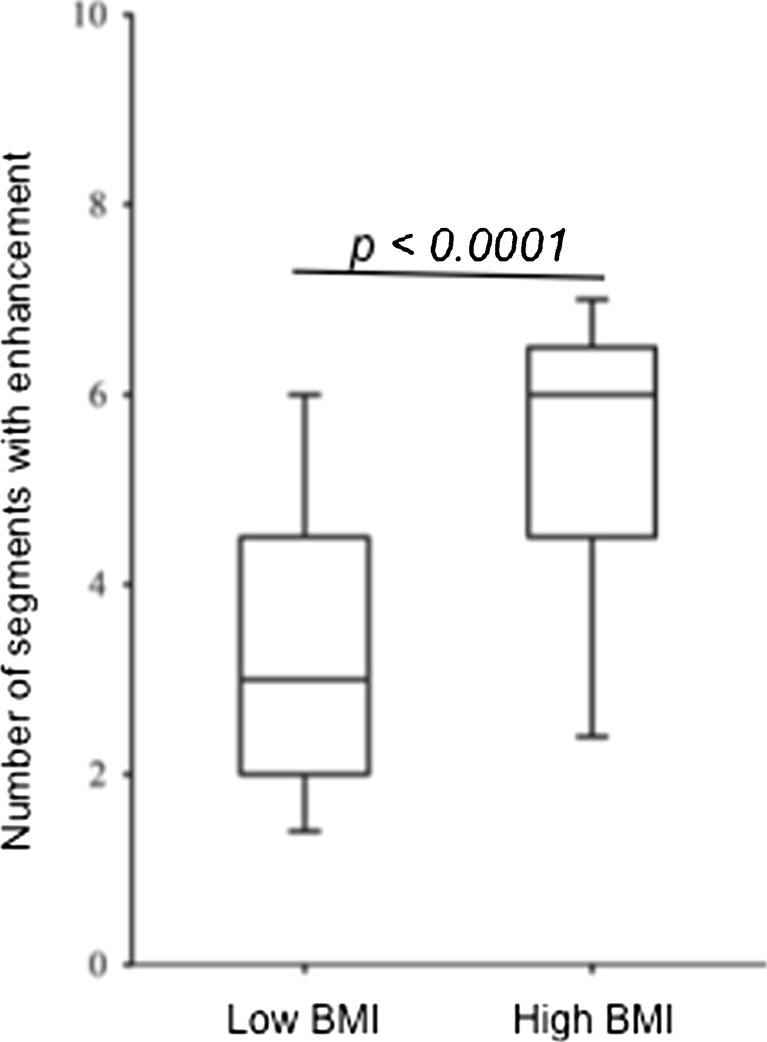
Comparison of number of coronary segments with enhancement after administration of the contrast agent. The group with the high BMI showed a significantly increased number of coronary segments per subject with gadolinium enhancement compared to the twin group with the low BMI

**Fig. 5:**
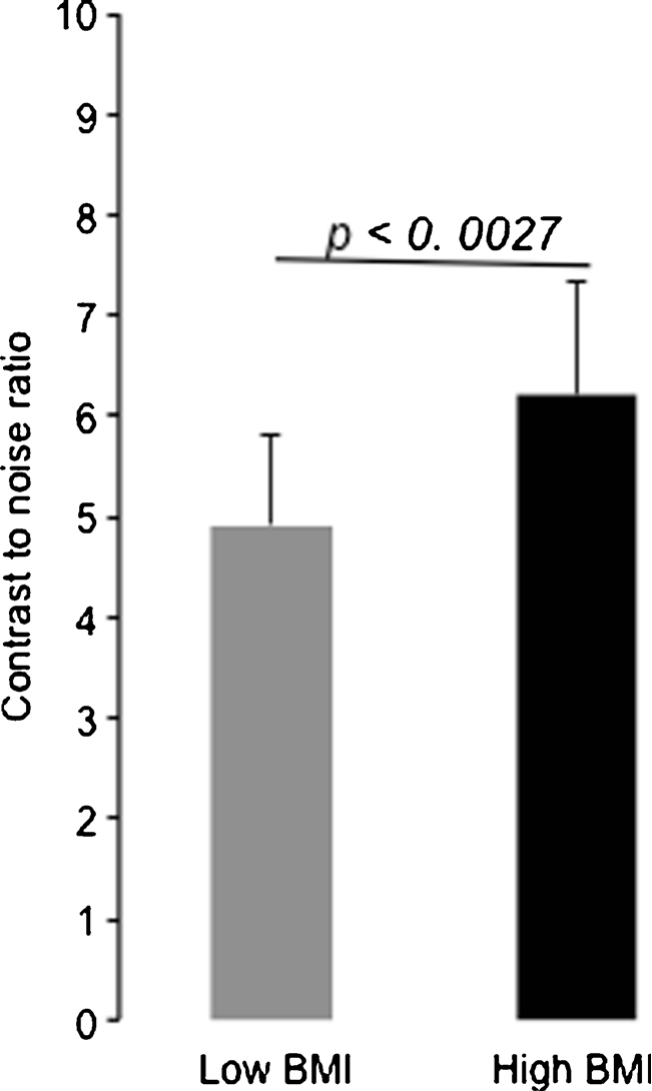
Comparison of coronary contrast to noise ratios (CNRs) after administration of the contrast agent. The group with the high BMI showed a significantly increased CNR following the administration of the contrast agent compared to the twin group with the low BMI. Values are shown as mean ± standard deviation

In 101 of 104 segments (97 %), in the twin group with the lower BMI, contrast enhancement within the coronary vessel wall was assessable (Figs. 3, 4 and 5). A small number of segments had to be excluded due to motion artefacts (n = 3). In 99 of 104 visible segments (95 %), in the twin group with the higher BMI, contrast enhancement within the coronary vessel wall was assessable (Figs. 3, 4 and 5). A small number of segments had to be excluded due to motion artefacts (n = 5).

No increase in signal intensity of the coronary vessel wall was measured prior to contrast administration in all twin pairs. Following the administration of the contrast agent, the twin group with the higher BMI demonstrated an increased number of enhancing coronary segments in absolute numbers and percentage per subject [5.3 ± 1.5 (66 ± 19 %), median 6.0, 25th percentile 4.5, 75th percentile

6.5] compared to the twin group with the lower BMI [3.5 ± 1.6 (44 ± 20 %), median 3.0, 25th percentile 2.0, 75th percentile 4.5; *p* < 0.0001]. Regarding the CNR, an increase in coronary vessel wall enhancement was measured in the high BMI twin group (6.1 ± 1.1) compared to the low BMI twin group (4.8 ± 0.9, *p* < 0.0027, n = 13 twin pairs).

### Interobserver and intraobserver variability

No significant bias was found between the two sets of repeated enhancement measurements (CNR +0.05 for intraobserver error and +0.35 for interobserver error) measured by Bland–Altman analyses. Intraobserver agreement was acceptable with 95 % limits of agreement ranging from 1.1 to -1.1. Overall, the interobserver 95 % limits of agreement were 2.01 to – 1.32.

## Discussion

This study in identical twins with significant differences in BMI demonstrated that an increase in BMI is associated with an increase in coronary vessel wall enhancement and the number of enhancing coronary segments. Due to the study design with monozygous twins, results are independent of genetic factors and can, therefore, be attributed to acquired factors predisposing to obesity, including lifestyle and environmental factors.

It is important to note that the study collective was limited to thirteen female identical twins with differences in BMI, which could be identified from the large TwinsUK Registry including more than 10,000 twin pairs. The fact that such a limited number of identical twin pairs could be identified, underlines the strong genetic influence on these two parameters. However, the design of our study is powerful as only monozygote twin pairs were investigated. Clear associations have been seen for other traits with these small subject numbers [22–25]. Regarding the sex of the investigated subjects, male and female subjects regularly differ in body fat distribution. It has been described that the regional distribution of body fat can be more important than excess adiposity per se in driving the risk for cardiovascular disease [26]. Therefore, the results of this investigation might not be applicable to the male population.

## Association of BMI with coronary artery disease

In this study, we could demonstrate that acquired obesity or an increase in BMI increases late gadolinium enhancement of the coronary arteries, potentially reflecting an increase in coronary atherosclerosis. From the genetical standpoint, obesity is a highly complex disorder that results in various health problems and an increase in morbidity and mortality [27]. It is recognized that complications of obesity are influenced by both, lifestyle factors and genetic predispositions. The influence of these two factors is difficult to differentiate in humans, especially in smaller patient collectives. Studies with monozygous twins discordant for obesity offer a unique opportunity to investigate the influence of these factors. Previous studies have shown that obesity is a highly heritable trait. However, environmental factors can influence the development of obesity [27]. With an increase in BMI, adipocytes start to accumulate at different sites of the body. Studies have indicated that in monozygous twins, the more obese twin demonstrates the highest increase in liver fat and intra-abdominal fat, and only a moderate increase in abdominal subcutaneous fat [28]. In our study, collective with monozygote female twin pairs with significantly different BMIs, the different groups are fully matched for genes. Such twin pairs are extremely difficult to identify. Our analysis resulted in only 13 obesity-discordant twin pairs from one nationwide twin cohort. This underlines the strong genetic component of the BMI.

The effects of obesity on atherosclerosis have also been investigated. It was shown that obesity is associated with a greater risk of coronary artery calcium and an increase in carotid artery intimal medial thickness. These associations persisted after adjustment for traditional cardiovascular disease risk factors [29]. Additionally, it was shown that a strong relationship between obesity and traditional cardiovascular disease risk factors exists [29]. The association between obesity and the risk for cardiovascular diseases is mediated by different possible pathways. Obesity was shown to affect glucose levels, lipid levels and blood pressure. These factors themselves were shown to contribute to the development of atherosclerosis. On a biochemical level, obesity is thought to lead to an increase in inflammatory factors and mediators which are expressed by the increased number of adipocytes [29].

## Non-targeted MR contrast agents for the assessment of the coronary wall

As the administration of non-targeted gadolinium-based contrast agents is approved for clinical use, this approach has been extensively tested in different patient collectives [8, 9, 30]. Nonspecific extracellular contrast agents, as used in this study, rapidly extravasate from the blood into the coronary vessel wall. This type of contrast agent was shown to accumulate in areas with delayed clearance, increased distribution volume or increased neovascularization [31–34]. T1 inversion recovery-based coronary vessel wall contrast-enhancement cardiovascular magnetic resonance (CE-CMR) sequences can be applied to visualize and quantify the uptake of the contrast agent into the atherosclerotic coronary wall. This approach has been validated in different patient studies and it was demonstrated that the area of coronary late enhancement correlates with the extent of coronary atherosclerosis [8, 9, 30]. _ENREF_20.

## Limitations:

This study collective was based on female twin pairs. Additionally, the overall number of twin pairs was limited. This was due to the rarity of identical twins with significant differences in BMI (13 out of more than 10,000) due to the strong genetic contribution and matching lifestyles. The average difference between identical twins is usually less than one kilogram. As only female twin pairs were investigated, the application of the results to the male population may be limited. The MR assessment of coronary wall enhancement was not validated by other invasive techniques in this study, e.g. intravascular ultrasound.

In conclusion, this study in monozygous twins indicates that acquired factors predisposing to obesity, including lifestyle and environmental factors, result in increased LGE of the coronary arteries, potentially reflecting an increase in coronary atherosclerosis in this female study population.

## Electronic supplementary material

Below is the link to the electronic supplementary material.

ESM 1 (DOCX 43 kb)
