# Erratum to: Influence of acquired obesity on coronary vessel wall late gadolinium enhancement in discordant monozygote twins

**DOI:** 10.1007/s00330-017-4884-y

**Published:** 2017-07-04

**Authors:** Marcus R. Makowski, Christian H. P. Jansen, Ullrich Ebersberger, Tobias Schaeffter, Reza Razavi, Massimo Mangino, Tim D. Spector, Rene M. Botnar, Gerald F. Greil

**Affiliations:** 1 Division of Imaging Sciences and Biomedical Engineering, King’s College London, London, UK; 2 Wellcome Trust and EPSRC Medical Engineering Centre, London, UK; 3 BHF Centre of Excellence, King’s College London, London, UK; 4 NIHR Biomedical Research Centre, King’s College London, London, UK; 5 Department of Radiology, Charité-Universitätsmedizin, Berlin, Germany; 6 Department of Cardiology and Intensive Care Medicine, Heart Center Munich-Bogenhausen, Munich, Germany; 7 Department of Twin Research and Genetic Epidemiology, King’s College London, London, UK; 8 National Institute for Health Research (NIHR) Biomedical Research Centre at Guy’s and St. Thomas’ Foundation Trust, London, UK

**Erratum to: Eur Radiol (2016) 1–7**

**DOI 10.1007/s00330-016-4616-8**

The article “Influence of acquired obesity on coronary vessel wall late gadolinium enhancement in discordant monozygote twins”, written by Marcus R. Makowski, Christian H. P. Jansen, Ullrich Ebersberger, Tobias Schaeffter, Reza Razavi, Massimo Mangino, Tim D. Spector, Rene M. Botnar, Gerald F. Greil, was originally published electronically on the publisher's internet portal (currently SpringerLink) on 14 October 2016 without open access.

As the study was partly supported by the Wellcome Trust, the article has been modified. The copyright of the article changed on 12 June 2017 to © The Author(s) [2016], and the article is forthwith distributed under the terms of the Creative Commons Attribution 4.0 International License (http://creativecommons.org/licenses/by/4.0/), which permits use, duplication, adaptation, distribution and reproduction in any medium or format, as long as appropriate credit is given to the original author(s) and the source, a link is provided to the Creative Commons license, and any changes made are indicated.

