# Supplementary material for: Influence of acquired obesity on coronary vessel wall late gadolinium enhancement in discordant monozygote twins
Source: Eur Radiol. 2016 Oct 14;27(11):4612–8. doi: 10.1007/s00330-016-4616-8 (PMC5635090; doi:10.1007/s00330-016-4616-8)
Supplement: Supplementary file 1 — (DOCX 43 kb) [file 330_2016_4616_MOESM1_ESM.docx]

**Short Form 36 (SF-36) Health Survey questionnaire**

This project was performed in cooperation with a research group working with the *BLINDED* Registry. Twins from the registry were recruited from the general population through national media campaigns in *BLINDED*. *BLINDED* Hospital research ethics committee approved the study (ethics number *BLINDED*), and informed consent was obtained from participating twins. Zygosity was established in all subjects by standardized questionnaire and was confirmed by DNA fingerprinting. Demographics, medical history and medication were available as part of the *BLINDED* cohort study. The risk factor the assessment in this study was based on the Short Form 36 (SF-36) Health Survey questionnaire (1–3). Details regarding the questionnaire can be found at www.twin-research.ac.uk.

**Definition of coronary segments**

For the analysis of the MRI, the coronary artery tree was broken down in eight segments after a modified American Heart Association (AHA) classification and all segments were analysed individually (4, 5).


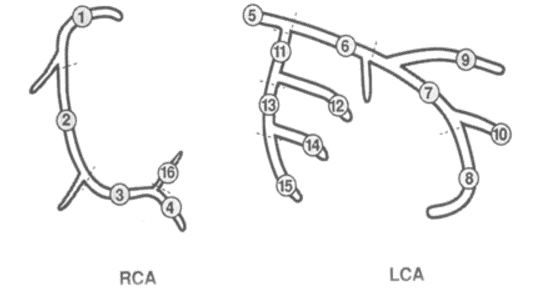


**Supplementary Figure 1:** Segmental anatomy of the coronary arteries after a modified American Heart Association classification. *1*, right coronary artery proximal; *2*, right coronary artery mid; *3*, right coronary artery distal; *4*, right posterior descendens; *5*, main stem; *6*, left anterior descending artery proximal; *7*, left anterior descending artery mid; *8*, left anterior descending artery distal; *9*, first diagonal; *10*, second diagonal; *11*, left circumflex artery proximal; *12*, obtuse marginal; *13*, left circumflex artery distal; *14*, left circumflex artery posterolateral branch; *15*, left circumflex artery posterodescendens branch; *16*, right coronary artery posterolateral branch. From (5, 6).

**References:**

1. Moayyeri A, Hammond CJ, Valdes AM, Spector TD. Cohort Profile: TwinsUK and healthy ageing twin study. Int J Epidemiol 2013;42:76–85.

2. Bowling A, Bond M, Jenkinson C, Lamping DL. Short Form 36 (SF-36) Health Survey questionnaire: which normative data should be used? Comparisons between the norms provided by the Omnibus Survey in Britain, the Health Survey for England and the Oxford Healthy Life Survey. J Public Health Med 1999;21:255–70.

3. Bowling A, Windsor J. Discriminative power of the health status questionnaire 12 in relation to age, sex, and longstanding illness: findings from a survey of households in Great Britain. J Epidemiol Community Health 1997;51:564–73.

4. Austen WG, Edwards JE, Frye RL et al. A reporting system on patients evaluated for coronary artery disease. Report of the Ad Hoc Committee for Grading of Coronary Artery Disease, Council on Cardiovascular Surgery, American Heart Association. Circulation 1975;51:5–40.

5. Ibrahim T, Makowski MR, Jankauskas A et al. Serial contrast-enhanced cardiac magnetic resonance imaging demonstrates regression of hyperenhancement within the coronary artery wall in patients after acute myocardial infarction. JACC Cardiovasc Imaging 2009;2:580–8.

6. Kaiser C, Bremerich J, Haller S et al. Limited diagnostic yield of non-invasive coronary angiography by 16-slice multi-detector spiral computed tomography in routine patients referred for evaluation of coronary artery disease. Eur Heart J 2005;26:1987–92.
